# Proteomic Identification of Plasma Components in *Tachypleus tridentatus* and Their Effects on the Longitudinal Bone Growth Rate in Rats

**DOI:** 10.3390/md21020111

**Published:** 2023-02-03

**Authors:** Shu Jiang, Xinjian Qu, Siping Liu, Jun Wei, Xiangxi Yi, Yonghong Liu, Chenghai Gao

**Affiliations:** 1 Institute of Marine Drugs, Guangxi University of Chinese Medicine, Nanning 530200, China; 2 Guangxi Key Laboratory of Marine Drugs, Nanning 530200, China

**Keywords:** *Tachypleus tridentatus*, longitudinal bone growth, transcriptomics, proteomics

## Abstract

*Tachypleus tridentatus* (*T. tridentatus*) is a marine animal and traditional Chinese medicine. *T. tridentatus* plasma is a valuable resource for important medical and health-based functions. In this experiment, in order to evaluate the effect and mechanism of *T. tridentatus* plasma with respect to the promotion of bone tissue growth in rats, the processes of ultrafiltration and mass spectrometry were first used to separate and identify the components of *T. tridentatus* plasma. Then, a comparison of the effects of the *T. tridentatus* plasma samples, which each possessed different molecular weights, regarding the growth of the long bones of rats was conducted. Finally, transcriptomics, proteomics, and bioinformatics were all used to analyze the biological functions and related signaling pathways of the *T. tridentatus* plasma in order to promote rat bone growth. The results showed that the contents of amino acid residues in peptides are related to the growth promotion that was contained in the 10–30 kDa plasma group. Moreover, the *T. tridentatus* plasma samples were found to be higher in this respect than those in the whole plasma group. In addition, the 10–30 kDa plasma group could significantly promote bone growth activity in rats. The proteomic analysis showed that the proteins that were differentially expressed in the 10–30 kDa plasma group were mainly enriched in the PI3K-AKT signal pathway. Our study suggested that the *T. tridentatus* plasma possesses promising potential for the purposes of clinical use, whereby it can serve the role of a growth-promoting agent.

## 1. Introduction

*Tachypleus tridentatus* (*T. tridentatus*) is a marine animal and traditional Chinese medicine [1]. *T. tridentatus* possesses anti-inflammatory, analgesic, hemostatic, and antidysentery medicinal effects [2,3,4]. It is also worth noting that *T. tridentatus* blood that contains hemocyanin possesses important medical application value [5]. The blood extract can be made into a Limulus-reagent (LR), which is widely used for the purposes of endotoxin detection and quantification in injections, as well as in radiopharmaceuticals, vaccines, and other biological products [2,3,6]. Indeed, Tachypleus amebocyte lysate (TAL) can be used for the purpose of accurate and rapid quantitative determination regarding bacterial infections or mycotoxins [7]. Most commonly in recent years, the developing COVID-19 vaccines have increased the demand for LR. Therefore, with such facts in mind, *T. tridentatus* blood is an important and irreplaceable resource. 

Short stature refers to a child’s height that is lower by more than two standard deviations in comparison to the normal height of individuals of the same race and in a similar environment [8]. Furthermore, the overall body size of mammals is mainly determined by longitudinal bone growth [9]. However, having said this, longitudinal bone growth is also the result of chondrocyte proliferation, as well as the ensuing hypertrophy in growth plates, which are called endochondral ossification and is mainly controlled by a system of the growth hormone-(GH-) insulin-like growth factor-1(IGF-1) axis [10,11]. The most important treatment for patients who possess a short stature is GH injection. However, daily injections and the abuse of GH in order to increase height, as well as the resulting pain that occurs, have drawn increasing attention in regard to medical ethics [12,13,14]. Shorter people have been reported to possess a lower health-related quality of life in adulthood, even though they no longer fall within the definition of short stature [15]. Therefore, a health product that can promote growth and development has become the top priority of many patients who possess a short stature.

*T. tridentatus* is one of the main sources of LR. However, less than 1% of *T. tridentatus* blood components have been used as fungal diagnostic reagents. In addition, more than 99% of the remaining plasma is discarded, thereby resulting in a great waste of resources [16]. In order to make full use of the remaining plasma, in this paper, the components remaining in the plasma are first separated and identified when compared to the effects of each molecular weight fragment of *T. tridentatus* plasma sample on the growth of long bones in rats, as well as then explored with respect to the mechanism by which the *T. tridentatus* plasma promotes bone growth in rats. This research not only helps to lay a theoretical foundation for the development of *T. tridentatus* plasma products but also helps to promote the high-value scientific utilization of plasma resources.

## 2. Results

### 2.1. T. tridentatus Plasma Ultrafiltration and HPLC Analysis 

A total of 256.52 g of solid powder samples were obtained as whole plasma (WP) samples after the lyophilization from 20 L of *T. tridentatus* plasma. 

Five samples were, in turn, obtained by separating 100 L of *T. tridentatus* plasma with 30 kDa, 10 kDa, 5 kDa, and 2 kDa ultrafiltration membrane modules. The samples were lyophilized in order to yield samples with molecular weights of 0–2 kDa, 2–5 kDa, 5–10 kDa, 10–30 kDa, and >30 kDa—which weighed 13.59 g, 16.50 g, 21.31 g, 10.64 g, and 155.56 g, respectively. The sample of *T. tridentatus* weighing >30 kDa plasma possessed the highest yield at 60.39%. Furthermore, the yields of the 0–2 kDa, 2–5 kDa, and 5–10 kDa samples were 20.80%, 6.41%, and 8.27%, respectively. Additionally, the lowest yield was obtained for the 10–30 kDa sample, which accounted for only 4.13% (Table 1).

**Figure 1:**
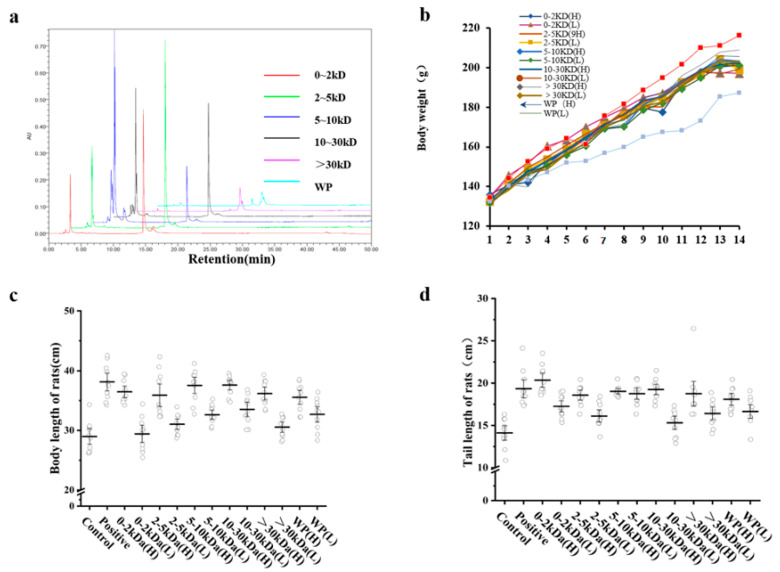
The effects of the *T. tridentatus* plasma on the growth performance of Wistar rats. (**a**) Chromatogram of the different components in the *T. tridentatus* plasma; (**b**) weight gain curve; (**c**) body lengths of the rats during the 14 d of sample administration; and (**d**) tail length of the rats during the 14d of sample administration. (*n* = 10 for each group, as determined by conducting one-way ANOVA; Control: saline group; and positive: recombinant human growth hormone group).

Each sample of *T. tridentatus* was collected and analyzed via high-performance liquid chromatography. With respect to this, the results showed that all six samples possessed peaks at *t*_R_ = 14.6 min, thereby indicating that the samples contained the same component (Figure 1a).

The candidate proteins of each sample were identified by protein mass spectrometry (MS). Further, the results showed that the >30 kDa sample and WP sample were mainly composed of hemocyanin subunits, while the <30 kDa samples were mainly composed of α-2 macroglobulin and complement-3-like proteins. The contents of arginine, valine, methionine, and hydrophobic amino acid residues in the peptides, which are related to growth-promoting activity contained in the 10–30 kDa sample, were found to be higher than those found in the WP sample (Table S1). 

### 2.2. The Effects of T. tridentatus Plasma on the Growth Activity Performance of Wistar Rats

In order to detect the effect of each *T. tridentatus* plasma sample on the growth and activity performances, Wistar rats were injected subcutaneously with rhGH 200 μg/kg in the positive control group (positive), and an equal volume of saline was injected into the rats in the negative control group (control). The high-dose experimental group was given each sample solution at a dosage of 200 mg/kg/d, and the low-dose experimental group was given each sample solution at a dosage of 100 mg/kg/d.

Changes in body weight, body length, and tail length are intuitive indicators of rat growth and development. On the 12th day, when compared with the control group, there was a significant difference in the body weight of the rats in each group (*p* < 0.05) (Figure 1b).

Furthermore, the body length results showed that the body length of each animal in the high-dose group was significantly different from that of the control group (Figure 1c). Moreover, among the experimental groups, the rats in the 10–30 kDa group possessed the longest body length (*p* < 0.05). When compared with the control group, the tail length results showed that there were significant differences in the tail lengths of the rats in each high-dose group, except in regard to the >30 kDa group. Furthermore, the tails of the rats in each low-dose group were shorter than those in the corresponding high-dose group (Figure 1d). In conclusion, the body weight, body length, and tail length of the rats were positively correlated with the concentration of each sample.

### 2.3. Effects of T. tridentatus Plasma on Bone Growth in Wistar Rats

**Figure 2:**
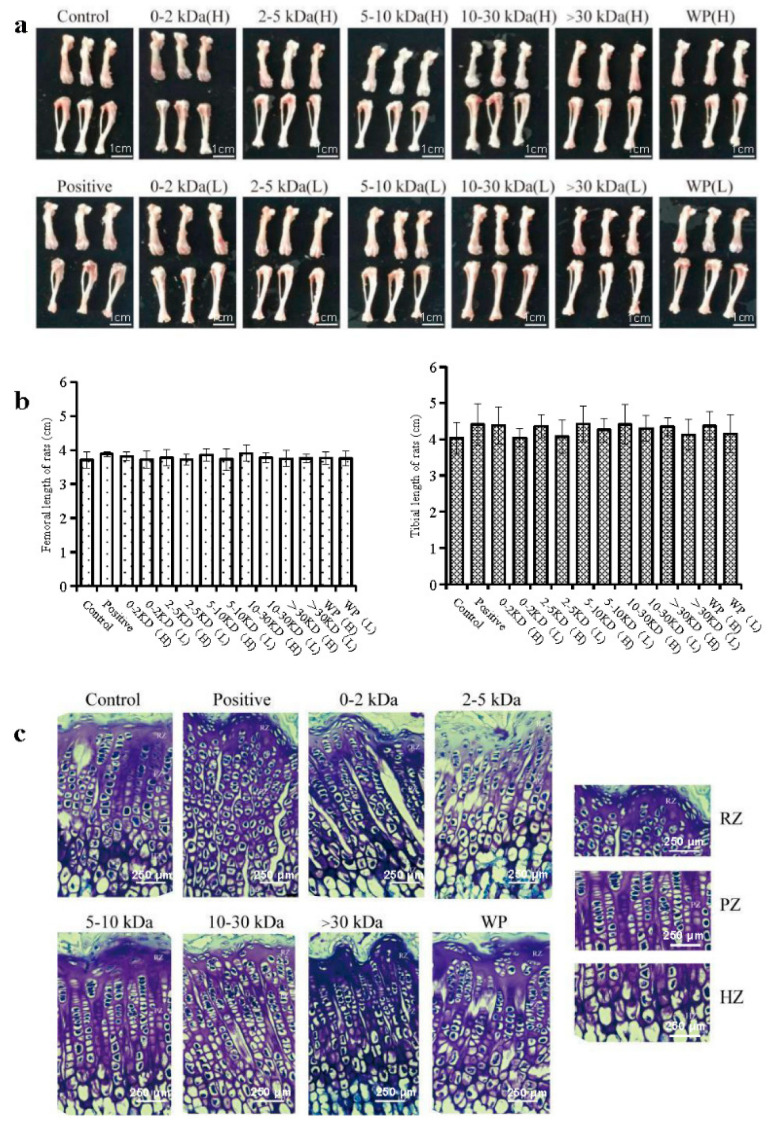
*T. tridentatus* plasma samples promoted normal skeletal growth in the rats after 14 d of subcutaneous injection. (**a**) Representative images of the tibial and femurs of rats, thereby demonstrating their length after 14 d of treatment. The scale bar is 1 cm; (**b**) the length of the femur and tibia in rats; and (**c**) photographs of cresyl violet-stained chondrocytes in the proximal growth plates of the tibias that were obtained from the control, positive (200 μg/kg), and *T. tridentatus* plasma-treated (200 mg/kg/d) rats. (Nonparametric test; RZ: resting zone; PZ: proliferative zone; HZ: hypertrophic zone; and scale bar = 250 μm). Each data point represents one individual rat. All data are shown as the mean ± standard deviation (s.d.). The scale bar is 250 μm.

Generally, changes in the length of the femur and tibia are indicators that directly reflect the growth of long bones in rats. As such, in order to avoid the influence of water and excess muscle tissue in regard to bone weight, the right femur of each rat was weighed. According to the results, the femur and tibia lengths of the rats in the 10~30 kDa (H) group were the longest (*p* > 0.05) (Figure 2a,b). Furthermore, the growth of long bones in rats is mainly accomplished by endochondral ossification, which is the development of growth plates in the metaphysis of the long diaphysis. 

The cresyl violet staining results with respect to the growth plate of the distal femur in the different groups of rats are shown in Figure 2c and Table 2. In the control group, the boundaries of the growth plates were clear; further, the cells were arranged in regular columns. However, as shown in Table 2, the height of the growth plate and the proliferative zone in the WP (H) and 30 kDa (H) groups were significantly higher than those in the control group (*p* < 0.05). Moreover, the animals in the 2–5 kDa, 5–10 kDa, and 10–30 kDa groups showed significantly increased growth plate heights than those found in the control group (*p* < 0.01).

### 2.4. Effects of the T. tridentatus Plasma on the Expression Levels of Serum ALP, BMP-2, and IGF-1 in Rats

The alkaline phosphatase (ALP) protein content detection in serum showed that, when compared with the control group, the positive group, 0–2 kDa (H) group, 2–5 kDa (H) group, 5–10 kDa (H) group, 10–30 kDa (H) group, >30 kDa (H) group, and the WP (H) group possessed significantly increased ALP contents in serum (*p* < 0.05).

When compared with the control group, the 0–2 kDa (H) group, 2–5 kDa (H) group, 5–10 kDa (H) group, and the 10–30 kDa (H) group, a significantly increased serum with bone morphogenetic protein-2 (BMP-2) concentrations (*p* < 0.01)was shown. In addition, the 0–2 kDa (L) group, 2–5 kDa (L) group, 5–10 kDa (L) group, >30 kDa (H) group, WP(H) group, and WP (L) group also showed increased BMP2 expression levels in serum (*p* < 0.05). The serum level of BMP-2 with respect to the rats in the 10–30 kDa (H) group was found to be the highest.

**Figure 3:**
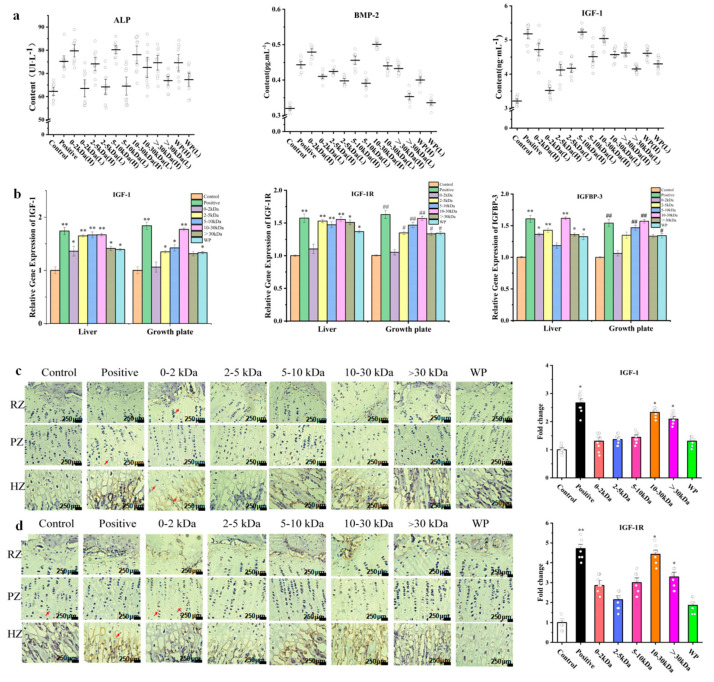
Different plasma components increased the factors related to growth and development in adolescent female rats. (**a**) Effects of different plasma components on total serum ALP, BMP-2, and IGF-1 levels in adolescent rats; and (**b**) quantification of the IGF-1, IGF-1R, and IGFBP-3 mRNA levels in the livers and growth plates that were obtained from the control, positive (200 μg/kg), and *T. tridentatus* plasma-treated (200 mg/kg/d, 100 mg/kg/d) rats (as determined via one-way ANOVA, *n* = 5 for each group). Each data point represents one individual rat. All data are shown as the mean ± standard deviation (s.d.). ^#^ *p* < 0.05 and ^##^ *p* < 0.01. (**c**) Representative immunohistochemical staining images of IGF-1 in the rat growth plates after 14 d of treatment; and (**d**) representative immunohistochemical staining images of the IGF-1R in the rat growth plates after 14 d of treatment (*n* = 10 for each group; nonparametric test; RZ: resting zone; PZ: proliferative zone; HZ: hypertrophic zone; and scale bar = 250 μm). * *p* < 0.05 and ** *p* < 0.01.

IGF-1 is a standard indicator for detecting bone growth. When compared with the control group, the 0–2 kDa (H) group, 5–10 kDa (H) group, 10–30 kDa (H) group, 10–30 kDa (L) group, >30 kDa (H) group, WP (H) group, and the positive group, significantly increased concentrations of IGF-1 in serum (*p* < 0.01) were observed. Moreover, the 2–5 kDa (H) group, 2–5 kDa (L) group, >30 kDa (L) group, 5–10 kDa (L) group, and WP (L) group showed increased concentrations of IGF-1 in serum (*p* < 0.05). Lastly, with respect to the aforementioned, there was no significant difference between the 0–2 kDa (L) group and the control group (Figure 3a). 

### 2.5. Effects of T. tridentatus Plasma on the Expression of Bone Growth-Related Genes in Rats

Quantitative RT-PCR was used in order to detect the mRNA expression levels of IGF-1, insulin-like growth factor-1 receptor (IGF-1R), and insulin-like growth factor binding protein 3 (IGFBP-3) in the Wistar rat tibial growth plates and livers (Figure 3b). When compared with the control group, the expression levels of IGF-1 mRNA in the liver of the rhGH group, 2–5 kDa group, and 10–30 kDa group were significantly increased (*p* < 0.01); moreover, the 5–10 kDa group, >30 kDa group, and the WP group also showed higher expressions than the control group (*p* < 0.05). In regard to the rat epiphyseal growth plate, the 2–5 kDa group, the 5–10 kDa group, and the WP group, an upregulated expression of IGF-1 mRNA (*p* < 0.05) was shown. 

When compared with the control group, the expression level of IGF-1 mRNA in the liver of the rhGH group, 2–5 kDa group, and 10–30 kDa group was significantly increased (*p* < 0.01). In addition, the 5–10 kDa group, >30 kDa group, and WP group also showed higher expressions than the control group (*p* < 0.05). In regard to the rat epiphyseal growth plate, the 2–5 kDa group, the 5–10 kDa group, and the WP group upregulated expression of IGF-1 mRNA (*p* < 0.05) was shown. 

In the rat livers, when compared with the control group, the rhGH group, 2–5 kDa group, and the 10–30 kD group showed a significantly upregulated mRNA expression of IGFBP-3 (*p* < 0.01). Regarding the 0–2 kDa group, >30 kDa group, and the WP group, increased expression levels (*p* < 0.05) were also shown. With respect to the epiphyseal growth plate-when compared with the control group- the rhGH group, 5–10 kDa group and the 10–30 kDa group displayed a significantly increased expression level of IGFBP-3 mRNA (*p* < 0.01). Lastly, the 2–5 kDa group and the WP group also showed increased IGFBP-3 mRNA expression levels (*p* < 0.05).

### 2.6. Effects of the T. tridentatus Plasma on the Expression of Cartilage-Related Proteins in Rat Growth Plates 

An immunohistochemical method was used to detect the expression of cartilage-related proteins in rat growth plates after 14 days of plasma treatment. Moreover, the IGF-1 protein is mainly expressed in chondrocytes in the hypertrophic zone, which are present at the base of the growth plate cartilage and around the trabecular bone (Figure 3c). When compared with the rats in the control group, the rats in the positive group, >30 kDa (H) group, and the 10–30 kDa (H) group showed a significantly upregulated expression of IGF-1 in the proximal tibia tissue (*p* < 0.05). 

The IGF-1R protein is expressed in the growth plate matrix, growth plate proliferation zone, and hypertrophy zone of the proximal tibia. When conducting an immunohistochemical analysis, the IGF-1R protein appeared brown (Figure 3d). The results demonstrated that compared with the control group, the rats in the rhGH group possessed a significantly increased expression level of IGF-1R protein (*p* < 0.01), while the animals in the 10–30 kDa and >30 kDa group displayed an upregulated expression of the IGF-1R protein (*p* < 0.01). Having said this there was no significant difference in the expression of this protein between the 0–2 kDa, 2–5 kDa, and 5–10 kDa samples when compared with the control group (*p* > 0.05).

### 2.7. The Femur Transcriptome of the Rats in the WP and 10–30 kDa Groups

**Figure 4:**
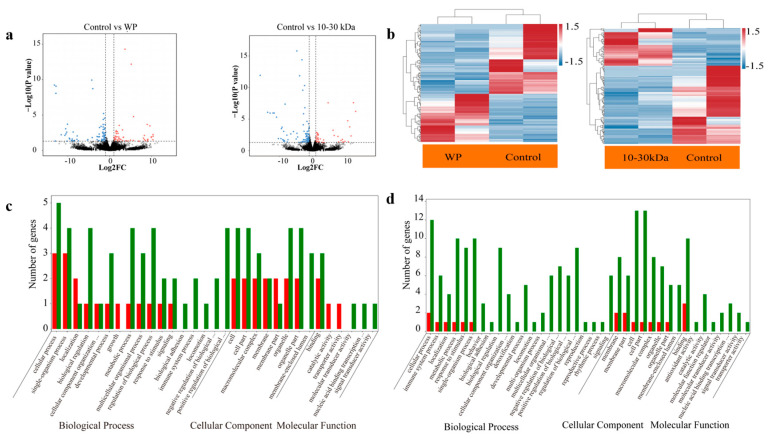
Femur transcriptomes, as determined by RNA sequencing. (**a**) RNA from the rats in the purified whole plasma and the 10–30 kDa group were assessed by RNA-seq; further, the transcript abundances were compared with DEseq2. Transcripts are plotted based upon fold change (between whole plasma, control, and between the 10–30 kDa and control) and adjusted p value (significance of differential expression), with mRNAs shown in lighter (±1 to 2.5 log2-fold change) or darker shades (>2.5 log2-fold change); (**b**) heatmap depicting the relative levels of genes in the whole plasma (left) and 10–30 kDa groups (right) (red represents relatively high expression, and blue represents relatively low expression); (**c**) GO analysis of the differentially expressed genes in the femur transcriptome of rats treated with WP for 14 d; and (**d**) GO analysis of the differentially expressed genes in the femur transcriptome of rats treated with the 10–30 kDa sample for 14 d. Red indicates the upregulated genes and green indicates the downregulated genes in the Go enrichment and classification histograms. Ontology covers cellular component, molecular function, and biological process domains.

When compared with the control group, the rats in the WP group had 130 differentially expressed genes (DEGs), of which 53 genes were upregulated, and 77 genes were downregulated, as shown in Figure 4a (left). The genes expression level in the WP group are shown in Figure 4b (left). When compared with the control group, 145 DEGs were identified in the 10–30 kDa group, including 47 upregulated genes and 98 downregulated genes, as shown in Figure 4a (right). The relative levels of gene expression in the 10–30 kDa group are shown in Figure 4b (right). As shown by the Gene Ontology (GO) analysis, the differential expression of the genes in the femur transcriptome of rats in the WP group, as well as the 10–30 kDa group, aggregated into biological processes and cellular components after 14 days of treatment (Figure 4c,d).

### 2.8. Quantitative Proteomics Analysis of Rat Femur Tissue in the WP and 10–30 kDa Groups

**Figure 5:**
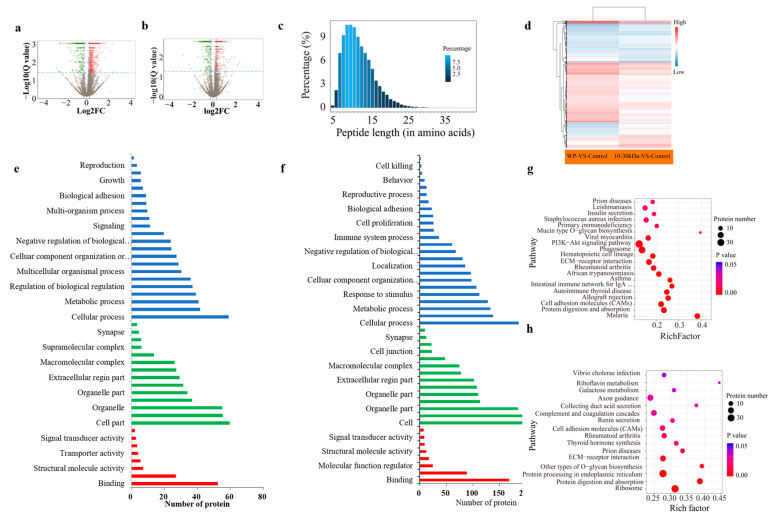
Comparative total proteomics of rats in the WP group and the 10–30 kDa group when compared with the control group. (**a**) Diagram of the total proteins from rat femur tissue in the purified WP group; (**b**) diagram of total protein from rat femur tissue in the purified 10–30 kDa group; (**c**) the percentage of each different peptide length; (**d**) clustering analysis of the DEPs in the comparisons of control group vs. 10–30 kDa group, as well as control group vs. the whole plasma group. Blue indicates downregulation, red indicates upregulation, and white indicates no detectable change in expression; (**e**) GO analysis of the DEPs for femur proteomics of the rats in the WP group; (**f**) GO analysis of the DEGs for femur proteomics of the rats in the 10–30 kDa group (blue indicates molecular function, green indicates cellular component, and red indicates biological process domains); (**g**) statistics of the pathway enrichment of the differentially expressed proteins in the control group vs. whole plasma group comparison; and (**h**) statistics of pathway enrichment of the differentially expressed proteins in the control group vs. the 10–30 kDa group comparison. RichFactor is the ratio of the number of differentially expressed proteins annotated in this pathway term with respect to the number of all proteins annotated in this pathway term.

The high-throughput identification and screening of differentially expressed proteins (DEPs) in the rat femur tissues of the rats in the WP and 10–30 kDa groups, was based on isotope labeling (iTRAQ) and proteomic technology. After filtering the data in order to exclude low-scoring spectra, 933,431 unique spectra-matching specific peptides were obtained. A total of 29,007 unique peptides were identified, thereby resulting in the identification of 5,860 proteins. A total of 388 DEPs were identified in the 10–30 kDa group when compared to the control group; in addition 926 DEPs were identified in the WP group when compared to the control group (Figure 5a,b). The numbers of DEPs and DEGs for these two comparisons are shown in Table 3.

### 2.9. The Femur Transcriptome of the Rats in the WP and 10–30 kDa Groups

All identified proteins and matching peptide lengths were analyzed and counted. Further, the results showed that most of the peptides were between seven and fifteen amino acid residues in length, which conformed to the rule of trypsin digestion with respect to peptides (Figure 5c). In addition, it must be noted that PRM is a new development in targeted mass spectrometry that is more specific and sensitive than selective response monitoring; it has been widely used for the quantification and detection of target proteins. Moreover, 15 candidate proteins related to long bone growth were screened for the purposes of PRM analysis, which converged with the protein expression levels measured by iTRAQ, thereby confirming that our iTRAQ results were reliable and reproducible (Figure S1).

The protein expression patterns were grouped for the purposes of cluster analysis whilst using Euclidean distance and hierarchical algorithms for the comparisons concerning the control group vs. 10–30 kDa group and control group vs. whole plasma group (Figure 5d). 

The functional analysis of the 220 DEPs in the WP group showed that most proteins acted on biological processes, whereby they accounted for 48.07% of the total proteins (Figure 5e). The GO analysis results of the 59 DEPs in the femur tissues of the rats in the 10–30 kDa plasma group were mainly enriched in molecular function, cellular component, and biological regulation. Among them, 14 proteins were enriched in the closed cavity of the cell membrane, six proteins were enriched in cell junctions, and six proteins were enriched in supramolecular complexes (Figure 5f). 

The functional classification of the DEPs in each comparison group was subjected to Clusters of Orthologous Groups of proteins (COG/KOG) analysis. The results showed that the functional types of DEPs in the WP group, as well as the 10–30 kDa, group were enriched in cell physiological processes, signal transduction mechanisms, post-translational modifications, protein conformational transitions, chaperones, etc. (Figure 5g,h). In the 10–30 kDa group, the enrichment of proteins related to intracellular transport, secretion transport, vesicular transport, extracellular structure-function, and information storage and processing was higher than those found in the WP group (Figure 5h).

### 2.10. Kyoto Encyclopedia of Genes and Genomes (KEGG) Pathway Enrichment Analysis of DEGs and DEPs 

Correlations between proteomes and transcriptomes are shown in the comparative proteomes and transcriptomes with respect to the comparisons of the control group vs. 10–30 kDa group, as well as the control group vs. the whole plasma group.

**Figure 6:**
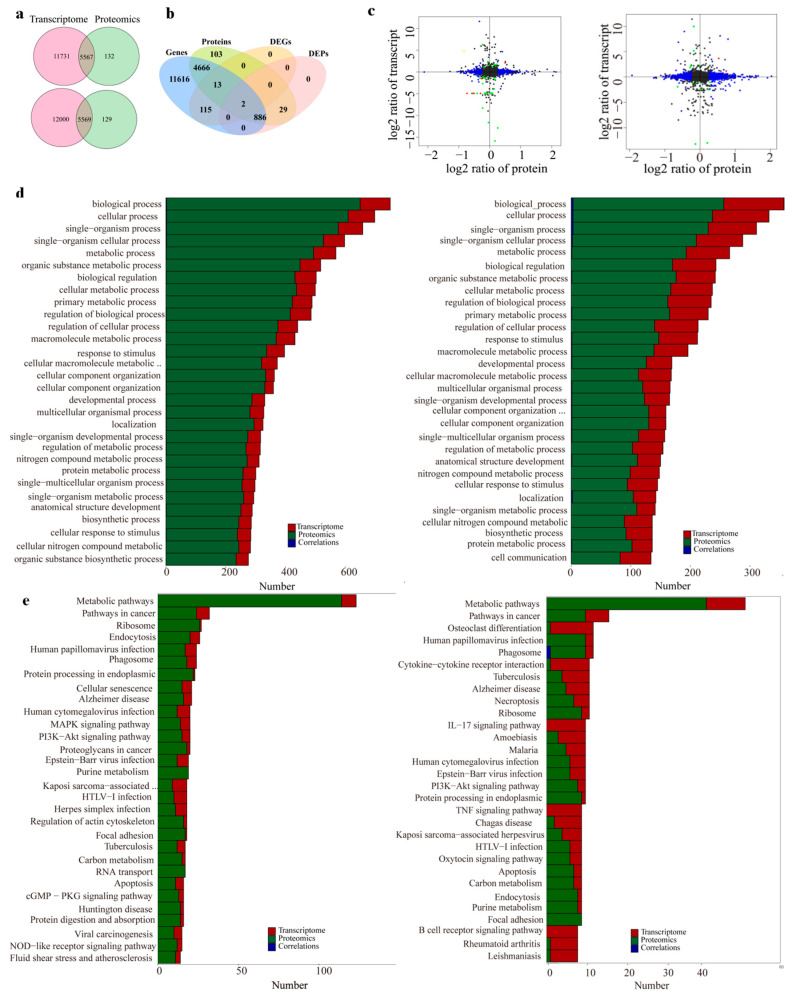
Combined proteomics and transcriptomics analyses. (**a**) Venn diagram of the expressed genes and proteins in the control group vs. the whole plasma group, as well as control group vs. the 10–30 kDa group comparisons; (**b**) comparison of all genes, proteins, DEGs, and DEPs in the control group vs. the whole plasma group (**left**), as well as control group vs. the 10–30 kDa group samples (**right**); (**c**) four-quadrant diagram of control group vs. the whole plasma group (**left**), as well as control group vs. the 10–30 kDa group (**right**) comparisons. (Each dot represents one gene/protein; black dots represent nondifferentiated proteins and genes; green dots represent DEGs but not the non-DEPs; blue dots represent DEPs but not the non-DEGs; and red dots represent both DEGs and DEPs with the same difference trend); (**d**) pathway analysis of the DEGs (**left**) and DEPs (**right**) from rat femurs; (**e**) Pathway correlation analysis in the control group vs. the whole plasma group (**left**), as well as control group vs. the 10–30 kDa group (**right**) comparisons.

The Venn diagram analysis found 5567 genes coexpressed in the control group vs. the whole plasma group, as well as 5569 coexpressed genes in the control group vs. the 10–30 kDa group (Figure 6a). Following a comparison of all the: genes; proteins; DEGs, and DEPs; P02688; D3ZC54; Q4V7F2; D3ZMS3; M0R9U2; Q5M7V3 and D4ACR1 showed the same trend with respect to the comparison of the transcriptomic and proteomic levels in the control group vs. the 10–30 kDa group (Figure 6b). In the comparison of the whole plasma group vs. the control group, there were only two DEGs/DEPs with the same expression trend at the transcriptomic and proteomic levels (Figure 6c). 

In order to determine the mode of action with respect to the samples in rats, the screened differential proteins were subjected to KEGG pathway analysis in order to determine the metabolic and signaling pathways involved. The KEGG signaling pathway analysis was performed on 220 differentially expressed proteins in the whole plasma group. In addition, a total of 44 related signaling pathways were retrieved; further, 16 signaling pathways were also significantly enriched. Among these, the main enrichment pathways were ribosome, endoplasmic reticulum protein processing, protein digestion, absorption, etc. (Figure 6d).

The KEGG signaling pathway analysis was performed on 59 DEGs in the 10–30 kDa group. Following this, a total of 43 related signaling pathways were enriched. The phosphatidylinositol 3-hydroxykinase/protein kinase B (PI3K-AKT) signaling pathway (ko04151), as well as the mitogen-activated protein kinase (MAPK) signaling pathway (ko04010) are most closely associated with the growth of long bones. Six pathways were enriched in both the comparisons of the control group vs. the 10–30 kDa group, as well as the control group vs. the whole plasma group (Figure 6e).

## 3. Discussion

*T. tridentatus* is an ancient marine arthropod whose evolutionary history can be traced back to approximately 450 million years ago; further, it is known as the famous living fossil [16]. Tachyplesin and Tatritin were isolated from the acidic extracts of *T. tridentatus.* In addition, they act as antimicrobial peptides with respect to the self-defense process in horseshoe crabs against invading microorganisms [4,17,18]. The *T. tridentatus* amoebocyte lysate is crucial in regard to medical testing [6], but its involvement in the industries of China in recent years has been limited due to the decline in the *T. tridentatus* population [5]. 

Ultrafiltration can separate polypeptides of different molecular weights while maintaining their physiological activities. Furthermore, the plasma proteomics of *T. tridentatus* is an important component of functional genome research, which is currently in its infancy. Most of the proteins identified are high-abundance proteins, such as hemocyanin, actin, α-2 macroglobulin, etc. The protein content in the *T. tridentatus* plasma is as high as 10% and is rich in 12 free amino acids [19]. The results of this study showed that hemocyanin was a highly abundant protein in the whole plasma of *T. tridentatus*. Furthermore, hemocyanin possesses various functions that are related to body energy storage, which is in addition to oxygen transport, the maintenance of osmotic pressure, and immune regulation [20]. Indeed, Alpha-2 macroglobulin is a macromolecular glycoprotein that is widely involved in the regulation and transport of substances in the Limulus plasma. With respect to this, certain studies have shown that α-2 macroglobulin is negatively correlated with the degree of chondrocyte apoptosis and thus can also be used as a carrier of growth factors in order to regulate the growth process of living cells [21].

*T. tridentatus* plasma can promote the growth and development of Wistar rats by increasing body weight, body length, and tail length in adolescent Wistar rats; in addition, despite this range of growth, the rats do not appear obese. In order to determine the effect of *T. tridentatus* plasma on longitudinal bone growth, we assessed macroscopic and biochemical growth-related factors in an experimental pubertal rat model. The mammalian growth plate consists of three principal zones: resting, proliferative, and hypertrophic zones. In regard to the resting zone, GH begins its actions by direct stimulation of resting stem-like chondrocytes in order to start proliferation [11,22]. The following cell replication in the proliferative zone, as well as with respect to the terminal differentiation and enlargement in the hypertrophic zone, are both primarily caused by circulatory IGF-1 and locally expressed IGF-1. Moreover, IGF-1 can promote bone reconstruction and improve the activity of osteoblasts [23]. In addition, BMP-2 can promote the growth of long bones and the development of epiphyseal cartilage, as well as play an important role in biological bone remodeling and bone formation [24,25]. Moreover, ALP is an important marker of osteoblasts and an important enzyme for the purposes of promoting osteoblast differentiation and mineralization [26,27,28]. Therefore, IGF-1, BMP2, and ALP are applied to monitor bone growth indicators. With respect to this, the plasma group showed macroscopic changes in total longitudinal bone length after 14 days of treatment. In addition, morphological changes were observed, such as the increased height of the growth plate area and an increased number of proliferating cells. Furthermore, *T. tridentatus* plasma treatment significantly increased the total serum levels of BMP-2, IGF-1R, and IGF-1, as well as induced the upregulated expression of IGF-1 and IGF-1R in the growth plate region. Taken together, these results suggest that *T. tridentatus* plasma extract can promote cell proliferation and longitudinal bone growth in the epiphyseal plate. 

In recent years, the use of proteomic techniques has emerged in the field of bone-related research. Based on iTRAQ, differential proteomics technology has been used in order to preliminarily screen the action pathway of the *T. tridentatus* whole plasma group, as well as the 10–30 kDa plasma group samples, in order to promote the growth of long bones in rats. In this study, it was found that the enrichment of proteins related to intracellular transport, secretion transport, vesicle transport, extracellular structure-function, as well as information storage and processing in the 10–30 kDa group was higher than those found in the whole plasma group.

There were examples of main enriched pathways among the differential proteins in the whole plasma group, including ribosome, endoplasmic reticulum protein processing, protein digestion, and protein absorption, as well as 20 related pathways, which were the main pathways involved in the 10–30 kDa plasma group. The differentially expressed proteins in the 10–30 kDa plasma group were mainly enriched in the PI3K-AKT signaling pathway, which were involved in the proliferation, differentiation, growth, and development of the pathway. Indeed, this pathway was speculated to be an important factor in terms of promoting bone tissue growth.

This study shows that there are still many active components in the plasma of *T. tridentatus,* such as hemocyanin and complement-3-like protein. This study establishes a research basis for the removal of high-abundance proteins from the *T. tridentatus* plasma and provides an experimental basis for the development of products that are related to the promotion of long bone growth.

## 4. Materials and Methods

### 4.1. Separation of T. tridentatus Plasma by Ultrafiltration

The process of *T. tridentatus* plasma ultrafiltration was performed on an ultrafiltration system with different membranes. In short, plasma was pumped with a peristaltic pump (bt100–2j, Lange Constant Flow Pump Co., Ltd. Baoding, China). All experiments were carried out with an ultrafiltration module (Bona Membrane Technology Co., Ltd., Jinan, China) in sequence with different nominal molecular weight cut-off values (i.e., 30, 10, 5, and 2 kDa) for the purposes of microporous polysulfone hollow fiber membranes (where the effective membrane area was 0.15 m^2^) [29]. Ultimately, the five fractions (0–2 kDa, 2–5 kDa, 5–10 kDa, 10–30 kDa, and >30 kDa) that were obtained from the *T. tridentatus* plasma were freeze-dried under vacuum at −50 °C, then severally stored at −20 °C.

### 4.2. HPLC Analysis of the Five Fractions

All sample solutions were filtered through a 0.45 μm membrane filter prior to analysis. The five fractions as described above were analyzed on a Waters 2695 Series HPLC System (Milford, MA, USA), which was equipped with a PDA detector and an Agilent 300 StableBond-C18 column (Palo Alto, CA, USA, 150 × 4.6 mm, 5 µm) at 30 ℃ with a flow rate of 1.0 mL/min. In addition, the mobile phase was composed of 0.1% trifluoroacetic acid (A) and acetonitrile (B) eluted with the following gradient: 0–10 min (5%), 10–15 min (5%–55%), 15–25 min (55%), 25–30 min (55%–100%), and 30–40 min (100%) solvent B. The injection volume was 10 µL, and the UV spectra were recorded at 260 nm.

### 4.3. UPLC-Q-Exactive Orbitrap Ms Analysis 

UPLC analysis was performed using an Ultimate 3000 system (Dionex, Sunnyvale, CA, USA), which was equipped with an online vacuum degasser, a quaternary pump, and an automatic sampler. Samples were separated with HYPERSIL GOLD C18 column (100 × 2.1 mm, 1.9 µm). Water that possessed 0.1% formic acid (A) and acetonitrile (B) was used as the mobile phase. Elution was carried out at a flow rate of 0.3 mL/min at 35 ℃ with the following gradient: 0–2 min, 2–5% (B); 2–5 min, 5–8% (B); 5–10 min, 8–15% (B); 10–15 min, 15–18% (B); 15–20 min, 18–40% (B); 20–24 min, 40–80% (B); 24–26 min, 80% (B); 26–27 min, 80–2% (B); and 27–30 min, 2% (B). 

### 4.4. Experimental Rats and Method of Administration

A total of 140 female Wistar rats (weighting 90 ± 10 g) aged 28 days were purchased from SJA Laboratory Animal Co., Ltd (Changsha, China). These 140 Wistar rats were randomly divided into 14 groups according to their body weight, with 10 rats in each group. The rats in the positive group were subcutaneously injected with 200 μg/kg of rhGH, and each treatment was given at high and low dosages of the solutions of the different molecular weight segments of T. tridentatus plasma. The high dosage subcutaneous injection was 200 mg/kg/d, while the low dosage was 100 mg/kg/d. The rats in the control group were subcutaneously injected with an equal volume of normal saline for a period of 14 consecutive days. All the experimental protocols and procedures that were conducted complied with the regulations of the Animal Ethics Committee of the Guangxi University of Traditional Chinese Medicine, as well as followed the Guidelines of Animal Care set by the Chinese Ministry of Health (GB/T 35823-2018).

### 4.5. Tissue Collection

After 5 days of acclimatization, the rats were randomly divided into the following fourteen groups (*n* = 10 rats in each group): control, recombinant human GH (rhGH) 200 μg/kg (positive group); 0–2 kDa 200 mg/kg (0–2 kDa (H) group); 0–2 kDa 100 mg/kg (0–2 kDa (L) group); 2–5 kDa 200 mg/kg (2–5 kDa (H) group); 2–5 kDa 100 mg/kg (2–5 kDa (L) group); 5–10 kDa 200 mg/kg (5–10 kDa (H) group); 5–10 kDa 100 mg/kg (5–10 kDa (L) group); 10–30 kDa 200 mg/kg (10–30 kDa (H) group); 10–30 kDa 100 mg/kg (10–30 kDa (L) group); >30 kDa 200 mg/kg (>30 kDa (H) group), >30 kDa 100 mg/kg (>30 kDa (L) group), whole plasma 200 mg/kg (WP (H) group); and the whole plasma 100 mg/kg (WP (L) group). The rats in the plasma treatment and positive groups were subcutaneously injected with different plasma fractions, or rhGH, once daily (8:30 a.m.) for 14 consecutive days. The control group accepted the hypodermic injection with an equivalent volume of normal saline. The body weight and food intake were recorded every day after starting the experiment. Finally, on the 15th day, the rats were sacrificed in order to collect the required tissues for the following experiments. Moreover, both tibias and femurs were removed surgically. 

### 4.6. Rat Growth Plate Height

Cresyl violet (CV) was purchased from Sigma-Aldrich (St. Louis, MO, USA), which can be used for chondrocyte staining to measure the height of growth plate. The heights of the overall growth plate, as well as the resting, proliferative, and hypertrophic zones, were measured at three different locations by ImageJ software. In addition, the proliferative zone (PZ) was measured from the flat chondrocytes aligned with the long axis of the bone that was presumed to be proliferative. The hypertrophic zone (HZ) was measured from chondrocytes that possessed nucleus and cytoplasm, which were easily distinguishable based on their size. The resting zone (RZ) was measured by subtracting the heights of the proliferative and hypertrophic zones from the overall height of the growth plate.

### 4.7. Enzyme-Linked Immunosorbent Assay (ELISA)

Regarding the quantitative analysis of the serum ALP, BMP-2, and IGF-1 concentrations, a sandwich assay was carried out, in duplicate, in a 96-well plate with an ELISA kit, according to the manufacturer’s protocol.

### 4.8. Immunohistochemistry

In order to detect ALP, BMP-2, and IGF-1 expression in the growth plate, the dehydrated sagittal sections of the tibia were pretreated as per the process described previously [30]. The sections were washed, treated with biotinylated rabbit antibody diluted at 1/200, and then incubated with an avidin-biotin complex reagent diluted at 1/100 for 1 h each. Sections were developed with a 0.05% 3,3-diaminobenzidine solution that contained hydrogen peroxide.

### 4.9. Real-Time Quantitative Polymerase Chain Reaction (PCR) Analysis

After sacrifice, the livers of the rats were promptly removed, rinsed, and stored at −80 °C. The total RNA from the liver samples was extracted using QIAzol reagent (Qiagen, Hilden, North Westphalia, Germany) and converted into cDNA with a transcription kit (Applied Biosystems, Foster City, CA, USA) according to the instructions. A quantitative PCR was performed on a real-time PCR system. The primers were designed and synthesized by Huiyuan Technology Co., Ltd. (Jinan, China). With respect to this, the primer sequences are shown in Table 4.

### 4.10. Transcriptomic Expression Profiling 

Total RNA was extracted using TRIzol reagent kit (Invitrogen, Carlsbad, CA, USA) according to the manufacturer’s protocol. RNA quality was assessed on an Agilent 2100 bioanalyzer (Agilent Technologies, Palo Alto, CA, USA) and checked using RNase-free agarose gel electrophoresis. After the total RNA was extracted, the eukaryotic mRNA was enriched with Oligo(dT) beads, while the prokaryotic mRNA was enriched by removing rRNA with a Ribo-ZeroTM Magnetic Kit (Epicentre, Madison, WI, USA). Then, the enriched mRNA was broken up into short fragments by utilizing a fragmentation buffer. Next, it was reverse transcribed into cDNA with random primers. The second-strand cDNA was synthesized by DNA polymerase I, RNase H, dNTPs, and a buffer. Then, the cDNA fragments were purified with a QiaQuick PCR extraction kit (Qiagen, Venlo, The Netherlands). Next, a base was added and then, finally, ligated to Illumina sequencing adapters. The ligation products were size selected via agarose gel electrophoresis, PCR amplified, and then sequenced using an Illumina Novaseq6000, by Gene Denovo Biotechnology Co. (Guangzhou, China).

### 4.11. Differential Gene Expression Analysis

In R software, the CPM value of the edgeR package was used for the purposes of correction, the genes with low expression in the samples were filtered, and the genes with RPKM values were output via using the RPKM function for the next analysis. The differentially expressed genes (DEGs) were identified through the limma package in R software. Furthermore, the genes meeting the cut-off criteria of logFC > 2.5 and FDR < 0.05 were regarded as DEGs. Moreover, the differentially expressed genes were visualized in volcano maps with the ggplot2 package (3rd edition) in R software (4.0).

### 4.12. Functional Annotation and Signaling Pathway Analysis

The GO and the KEGG analyses of the genes were conducted in order to study their main functions and participation with respect to the signaling pathways. In this paper, the clusterProfiler and enrichplot package in R software was used to explore the functions between the core genes and the involved signaling pathways. In addition, the ggplot2 package was used to visualize the output results.

### 4.13. Quantification of DEPs by PRM

In each group, a total of 15 DEPs were selected for the purposes of validation by PRM. Protein extraction and tryptic digestion were performed following the same protocol used for the label-free quantitative proteomics experiment.

### 4.14. Statistical Analysis

The statistical analysis and graphical representation of the data were carried out usingthe SPSS software, version 22.0 (SPSS Inc., Chicago, IL, USA). Further, a one-way analysis of variance was used for the purposes of multiple comparisons. The statistical significance was set at *p* < 0.05. All values were presented as mean ± SD.

## Figures and Tables

**Table 1:** Ultrafiltration separation of *Tachypleus tridentatus* plasma.

Ultrafiltration-Separated Samples	Molecular Weight (kDa)	Weight (g)	Proportion (%)
1	0–2	53.59	20.80
2	2–5	16.50	6.41
3	5–10	21.31	8.27
4	10–30	10.64	4.13
5	>30	155.56	60.39

**Table 2:** Height of overall growth plate, as well as the proliferative and hypertrophic zone in each group (*n* = 10).

Group	Overall Growth Plate (μm)	Proliferative Zone (μm)	Hypertrophic Zone (μm)
Control	95.31 ± 5.38	62.76 ± 2.18	29.56 ± 3.14
Positive	144.51 ± 2.92 **	96.32 ± 1.47 **	46.12 ± 2.81 **
0–2 kDa (H)	108.10 ± 7.01	73.44 ± 4.03	33.61 ± 1.76
2–5 kDa (H)	121.74 ± 3.89 **	83.07 ± 3.62 **	40.36 ± 3.71 *
5–10 kDa (H)	122.72 ± 6.73 **	83.76 ± 3.19 **	39.88 ± 6.66 *
10–30 kDa (H)	128.74 ± 4.02 **	87.05 ± 0.77 **	42.48 ± 3.62 *
>30 kDa (H)	113.99 ± 8.81 *	76.15 ± 1.04 *	35.84 ± 1.99
WP (H)	110.96 ± 4.62 *	75.12 ± 2.30 *	35.05 ± 3.21

Results represented as mean ± SD. * *p* < 0.05, and, ** *p* < 0.01 vs. control group.

**Table 3:** Numbers of DEPs and DEGs in the two comparisons.

Comparisons	Up-Regulated Expression Genes/Protein Number	Down-Regulated Expression Genes/Protein Number	Total Different ExPression Gene/Protein Number
10–30 kDa-vs-Control	47/214	98/174	145/388
Whole plasma-vs-Control	53/574	77/352	130/926

**Table 4:** Real-time primer sequence.

Gene	Primer Sequence
IGF-1 F	5′-GCTATGGCTCCAGCATTCG-3′
IGF-1 R	5′-TCCGGAAGCAACACTCATCC-3′
IGFBP-3 F	5′-GGAAAGACGACGTGCATTG-3′
IGFBP-3 R	5′-GCGTATTTGAGCTCCACGTT-3′
IGF-1R F	5′-GGTCTCTAAGGCCAGAGGTGGA-3′
IGF-1R R	5′-GACGAACTTGTTGGCATTGAGGTA-3′
β-actin F	5′-CGTTGACATCCGTAAAGAC-3′
β-actin R	5′-TAGGAGCCAGGGCAGTA-3′

## Data Availability

The data presented in this study are available upon request from the corresponding author.

## Supplementary Materials

The following supporting information can be downloaded at: https://www.mdpi.com/article/10.3390/md21020111/s1, Figure S1: The Comparison of protein expression by iTRAQ and PRM; Table S1: List of proteins from MS analysis; Table S2: Significantly different expressed proteins in the control vs. whole plasma comparison; Table S3: Significantly different expressed proteins in control vs. 10–30 kDa comparison.
