# Supplementary material for: Proteomic Identification of Plasma Components in Tachypleus tridentatus and Their Effects on the Longitudinal Bone Growth Rate in Rats
Source: Mar Drugs. 2023 Feb 3;21(2):111. doi: 10.3390/md21020111 (PMC9961754; doi:10.3390/md21020111)
Supplement: Supplementary file 1 [file marinedrugs-21-00111-s001.zip › marinedrugs-2180201-supplementary.pdf]

Supplementary Information

Proteomic Identification of Plasma Components in *Tachypleus tridentatus* and Their Effects on the Longitudinal Bone Growth Rate in Rats

Jiang S. *et al*.

This file includes Supplementary Figure S1, Table S1 and Table S2.

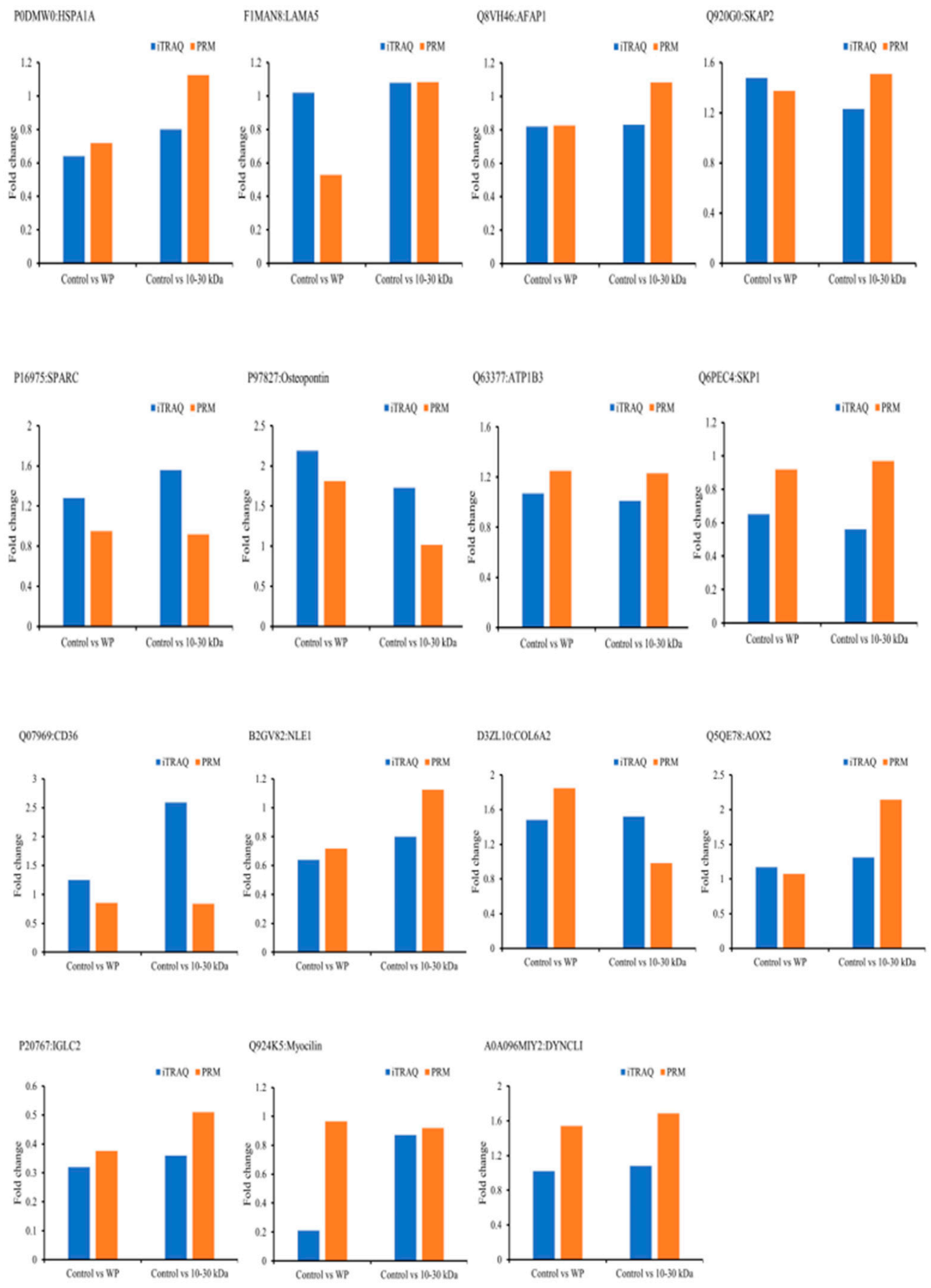

Figure S1. 15 candidate proteins related to long bone growth were screened for PRM analysis. The 15 candidates include HSPA1A, LAMA5,AFAP1, SKAP2, SPARC, Osteopontin, ATP1B3, SKP1, CD36, NLE1, COL6A2, AOX2, IGLC2， Myocilin and DYNCL1.

Table S2. Significant difference protein in control-VS-whole plasma samples

Protein Number	Protein Description	Average Ratio	P value
tr|A0A1W2Q6L0|A0A1W2Q6L0_RAT	Mediator complex subunit 14	1.51	0.01553
tr|D3ZZC1|D3ZZC1_RAT	RCG43947	1.51	0.009149
tr|Q9R1X8|Q9R1X8_RAT	PKC lambda protein	1.51	0.04923
tr|D3ZDP2|D3ZDP2_RAT	Mitochondrial ribosomal protein L58	1.51	0.006154
sp|P24594|IBP5_RAT	Insulin-like growth factor-binding protein 5	1.51	0.02528
sp|P59722|EGLN1_RAT	Egl nine homolog 1	1.51	0.0259
tr|B2RZD4|B2RZD4_RAT	60S ribosomal protein L34	1.51	0.04743
sp|P09650|MCPT1_RAT	Mast cell protease 1	1.52	0.01335
sp|P69478|CHSTB_RAT	Carbohydrate sulfotransferase 11	1.52	0.02526
tr|D4A9I7|D4A9I7_RAT	Leucine-rich repeat-containing 3B	1.52	0.03867
sp|Q9Z0W5|PACN1_RAT	Protein kinase C and casein kinase substrate in neurons protein 1	1.52	0.0002775
tr|B5DFK8|B5DFK8_RAT	Pdxdc1 protein	1.52	0.003978
sp|P02680|FIBG_RAT	Fibrinogen gamma chain	1.52	0.02739
tr|D3ZQQ2|D3ZQQ2_RAT	WIZ zinc finger	1.53	0.01944
tr|A0A0G2JYL4|A0A0G2JYL4_RAT	Prolyl 4-hydroxylase subunit alpha 2	1.53	0.01589
tr|Q2I6B0|Q2I6B0_RAT	V-type proton ATPase subunit a	1.53	0.01975
tr|A0A0H2UI04|A0A0H2UI04_RAT	Probable inactive glycosyltransferase 25 family member 3	1.53	0.01809
tr|D3ZRM3|D3ZRM3_RAT	Hexosyltransferase	1.53	0.01754
sp|Q6AYP7|5NT3B_RAT	7-methylguanosine phosphate-specific 5'-nucleotidase	1.53	0.04684
sp|Q8VH46|AFAP1_RAT	Actin filament-associated protein 1	1.53	0.009302
sp|Q5PQL5|PTSS1_RAT	Phosphatidylserine synthase 1	1.53	0.04091
sp|Q5FVL0|VA0D2_RAT	V-type proton ATPase subunit d 2	1.53	0.002971
sp|O88280|SLIT3_RAT	Slit homolog 3 protein GN=Slit3	1.53	0.02147
tr|Q5RJR9|Q5RJR9_RAT	Serine (Or cysteine) proteinase inhibitor, clade H, member 1	1.53	0.0009304
tr|O70509|O70509_RAT	CD44 antigen	1.54	0.002804
tr|A0A0G2JXC1|A0A0G2JXC1_RAT	Protein ERGIC-53	1.54	0.0005437
sp|P61751|ARF4_RAT	ADP-ribosylation factor 4	1.54	0.002999
tr|A0A0G2K7L2|A0A0G2K7L2_RAT	Immunoglobulin superfamily, member 3	1.55	0.003512
sp|Q9QYU2|EFTS_RAT	Elongation factor Ts, mitochondrial	1.55	0.00004569
tr|G3V803|G3V803_RAT	Cadherin-2	1.55	0.02242
sp|D3ZCL3|RU1C_RAT	U1 small nuclear ribonucleoprotein C	1.55	0.01143

tr|Q6AY18|Q6AY18_RAT	SAR1 gene homolog A	1.55	0.04424
sp|O70535|LIFR_RAT	Leukemia inhibitory factor receptor	1.55	0.01509
tr|Q6P7B6|Q6P7B6_RAT	Ephrin B1	1.55	0.01299
sp|P05426|RL7_RAT	60S ribosomal protein L7	1.56	0.03821
tr|F1M798|F1M798_RAT	Metalloendopeptidase	1.56	0.01024
sp|P14480|FIBB_RAT	Fibrinogen beta chain	1.56	0.01015
sp|Q9R0C5|GALT7_RAT	N-acetylgalactosaminyltransferase 7	1.56	0.001316
tr|D4ADP9|D4ADP9_RAT	Coatomer subunit zeta	1.56	0.002394
tr|D3Z9K6|D3Z9K6_RAT	Family with sequence similarity 180, member A	1.56	0.01033
sp|P23097|MMP13_RAT	Collagenase 3 (Fragment)	1.56	0.001485
tr|D4A6W6|D4A6W6_RAT	Ribosomal_L2_C domain-containing protein	1.56	0.01295
sp|P31211|CBG_RAT	Corticosteroid-binding globulin	1.57	0.04139
tr|A0A0G2K5T3|A0A0G2K5T3_RAT	Phosphatidylinositol 4,5-bisphosphate 3-kinase catalytic	1.57	0.02849
tr|A0A0G2JZS9|A0A0G2JZS9_RAT	Ig-like domain-containing protein	1.57	0.001081
tr|Q0QEW8|Q0QEW8_RAT	Ribosomal protein L18 (Fragment)	1.57	0.02815
tr|D3ZMR0|D3ZMR0_RAT	Olfactomedin-like 2A	1.58	0.003912
sp|P07897|PGCA_RAT	Aggrecan core protein	1.58	0.002414
sp|Q63377|AT1B3_RAT	Sodium/potassium-transporting ATPase subunit beta-3	1.58	0.0001596
tr|G3V7V5|G3V7V5_RAT	Peptidylprolyl isomerase	1.58	0.01484
tr|G3V6R4|G3V6R4_RAT	Leucine-rich repeat-containing 1	1.59	0.01318
tr|Q66H06|Q66H06_RAT	Tetraspanin	1.59	0.03541
tr|A0A292G0W2|A0A292G0W2_RAT	Gai-interacting protein	1.59	0.04169
sp|P24090|FETUA_RAT	Alpha-2-HS-glycoprotein	1.6	0.003106
sp|P61621|S61A1_RAT	Protein transport protein Sec61 subunit alpha isoform 1	1.6	0.00567
tr|Q63109|Q63109_RAT	Coagulation factor X (Fragment)	1.61	0.007705
sp|P07861|NEP_RAT	Neprilysin	1.61	0.007377
sp|Q9R1J8|P3H1_RAT	Prolyl 3-hydroxylase 1	1.61	0.00003234
tr|M0R3V4|M0R3V4_RAT	Myeloid-derived growth factor	1.62	0.03361
tr|A0A0G2K7C2|A0A0G2K7C2_RAT	Kinesin family member 20B	1.62	0.04472
tr|F1M853|F1M853_RAT	Ribosome-binding protein 1	1.62	0.0003963
sp|O35186|CATK_RAT	Cathepsin K	1.62	0.01286
tr|B0K010|B0K010_RAT	Thioredoxin domain-containing 17	1.63	0.02971
sp|P31720|C1QA_RAT	Complement C1q subcomponent subunit A	1.63	0.04097

tr|A0A0H2UHM3|A0A0H2UHM3_RAT	Haptoglobin	1.64	0.03299
tr|F1LSW7|F1LSW7_RAT	60S ribosomal protein L14	1.64	0.04956
sp|P07335|KCRB_RAT	Creatine kinase B-type	1.64	0.02534
tr|A0A0G2K7I1|A0A0G2K7I1_RAT	Ig-like domain-containing protein	1.65	0.04929
tr|D4A4L6|D4A4L6_RAT	Ig-like domain-containing protein	1.65	0.0002873
sp|Q5RK27|S12A7_RAT	Solute carrier family 12 member 7	1.66	0.0406
sp|Q4JM44|SMS2_RAT	Phosphatidylcholine:ceramide cholinephosphotransferase 2	1.66	0.006487
sp|P61314|RL15_RAT	60S ribosomal protein L15	1.66	0.01683
tr|G3V7K5|G3V7K5_RAT	NPC intracellular cholesterol transporter 1	1.67	0.006344
tr|F1LN88|F1LN88_RAT	Aldehyde dehydrogenase, mitochondrial	1.67	0.006583
tr|B1WBV5|B1WBV5_RAT	Epiphycan	1.67	0.03172
tr|D4A8L3|D4A8L3_RAT	Sphingomyelin phosphodiesterase 3	1.68	0.0004729
tr|A7M6E9|A7M6E9_RAT	Semaphorin 3D	1.68	0.001053
tr|A0A0G2K1L8|A0A0G2K1L8_RAT	Brain acid soluble protein 1	1.68	0.01871
sp|O35276|NRP2_RAT	Neuropilin-2	1.68	0.004991
tr|F1LVV3|F1LVV3_RAT	RAN-binding protein 9	1.68	0.03055
sp|P84100|RL19_RAT	60S ribosomal protein L19	1.68	0.0431
tr|D3ZP13|D3ZP13_RAT	Sulfhydryl oxidase	1.69	0.003651
tr|A0A0G2K2S2|A0A0G2K2S2_RAT	Solute carrier family 2, facilitated glucose transporter member 1	1.69	0.01126
sp|Q5BJP3|UFM1_RAT	Ubiquitin-fold modifier 1	1.69	0.001307
tr|Q7TQ70|Q7TQ70_RAT	Ac1873	1.7	0.01655
tr|D3Z9Z7|D3Z9Z7_RAT	Collagen beta(1-O)galactosyltransferase 2	1.7	0.005645
tr|D3ZF11|D3ZF11_RAT	Hepatitis B virus x interacting protein	1.71	0.04951
tr|A0A0G2JVA4|A0A0G2JVA4_RAT	Procollagen-lysine,2-oxoglutarate 5-dioxygenase 2	1.72	0.002331
sp|Q6AYF6|GL8D1_RAT	Glycosyltransferase 8 domain-containing protein 1	1.72	0.0004893
sp|O55164|MPDZ_RAT	Multiple PDZ domain protein	1.72	0.0002163
tr|D4A5Z6|D4A5Z6_RAT	Coiled-coil domain-containing 34	1.72	0.04846
tr|B5DEF3|B5DEF3_RAT	Gamma-glutamyl carboxylase	1.72	0.0108
sp|Q66H78|PXYP1_RAT	2-phosphoxylose phosphatase 1	1.73	0.03293
tr|F6T0B3|F6T0B3_RAT	Collagen type XI alpha 2 chain	1.73	0.00104
tr|M0R5U6|M0R5U6_RAT	Torsin-1A-interacting protein 2	1.74	0.0346
tr|D3ZWI0|D3ZWI0_RAT	Cell migration-inducing hyaluronidase	1.76	0.02429
tr|O88550|O88550_RAT	Caspase 7	1.76	0.04613

sp|Q62703|RCN2_RAT	Reticulocalbin-2	1.79	0.005079
sp|P61354|RL27_RAT	60S ribosomal protein L27	1.8	0.02577
tr|A0A0G2JX36|A0A0G2JX36_RAT	Ig-like domain-containing protein	1.8	0.002145
sp|P63182|CBLN1_RAT	Cerebellin-1	1.82	0.04584
tr|D4A275|D4A275_RAT	Glucoside xylosyltransferase 2	1.84	0.006836
tr|D3ZKR8|D3ZKR8_RAT	Protein kish	1.85	0.00003605
tr|A0A0G2K7A5|A0A0G2K7A5_RAT	Collagen type X alpha 1 chain	1.85	0.00172
sp|P16391|HA12_RAT	RT1 class I histocompatibility antigen	1.85	0.002267
sp|Q62632|FSTL1_RAT	Follistatin-related protein 1	1.86	0.006747
tr|Q5U2V1|Q5U2V1_RAT	Peptidylprolyl isomerase	1.86	0.00125
sp|P08289|PPBT_RAT	Alkaline phosphatase, tissue-nonspecific isozyme	1.86	0.0002146
sp|O08776|NDUF3_RAT	NADH dehydrogenase1 alpha subcomplex assembly factor 3	1.87	0.01613
sp|P97586|CGRE1_RAT	Cell growth regulator with EF hand domain protein 1	1.87	0.001057
tr|F1LNY3|F1LNY3_RAT	Neural cell adhesion molecule 1	1.88	0.002889
tr|F1LPB5|F1LPB5_RAT	kDEL endoplasmic reticulum protein retention receptor 3	1.88	0.03893
tr|D3ZH41|D3ZH41_RAT	Cytoskeleton-associated protein 4	1.9	0.0005229
sp|Q9WVC1|SLIT2_RAT	Slit homolog 2 protein	1.91	0.002245
tr|F1M7F7|F1M7F7_RAT	Complement component C6	1.94	0.01856
tr|D3ZQP6|D3ZQP6_RAT	Sema domain, immunoglobulin domain (Ig), and GPI membrane anchor	1.94	0.008988
tr|A0A096P6L8|A0A096P6L8_RAT	Fibronectin	1.94	0.001105
sp|Q6AY25|TMED3_RAT	Transmembrane emp24 domain-containing protein 3	1.95	0.0001436
sp|Q8CG08|CTHR1_RAT	Collagen triple helix repeat-containing protein 1	1.95	0.0004893
tr|G3V723|G3V723_RAT	Phosphate regulating gene with homologies to endopeptidases on the X chromosome	1.95	0.0009709
tr|Q4FZY1|Q4FZY1_RAT	Cd68 molecule	1.95	0.02132
sp|P62083|RS7_RAT	40S ribosomal protein S7	1.97	0.02133
tr|D4ADS6|D4ADS6_RAT	Integrator complex subunit 7	1.97	0.02723
sp|Q5PQX0|UXS1_RAT	UDP-glucuronic acid decarboxylase 1	1.98	0.0177
tr|D3Z9M5|D3Z9M5_RAT	Peptidylprolyl isomerase	1.98	0.004331
tr|A0A0G2JSM1|A0A0G2JSM1_RAT	Ratsg2	1.99	0.0001051
tr|D4A4H5|D4A4H5_RAT	Stromal cell derived factor 2	2	0.007248

sp|Q9EPH1|A1BG_RAT	Alpha-1B-glycoprotein	2.1	0.0003004
tr|A0A096MJU4|A0A096MJU4_RAT	Solute carrier family 37 member 2	2.13	0.01224
tr|A0A0G2K4I9|A0A0G2K4I9_RAT	Coagulation factor XI	2.18	0.00006485
tr|A0A0G2K9Y0|A0A0G2K9Y0_RAT	Immunoglobulin heavy constant mu	2.19	0.003542
tr|D3ZKU5|D3ZKU5_RAT	Uncharacterized protein	2.21	0.02498
tr|G3V7N9|G3V7N9_RAT	Adiponectin a	2.27	0.03887
tr|A0JPL7|A0JPL7_RAT	Leukocyte cell-derived chemotaxin 1	2.28	0.008315
sp|P20909|COBA1_RAT	Collagen alpha-1(XI) chain	2.28	0.0004749
tr|B1WBR1|B1WBR1_RAT	Golgi reassembly-stacking protein 1	2.31	0.006278
sp|Q6IE14|TM11L_RAT	Transmembrane protease serine 11B-like protein	2.34	0.0009055
tr|Q6IMK1|Q6IMK1_RAT	Sp7 transcription factor	2.41	0.02133
tr|D3ZFK6|D3ZFK6_RAT	Autophagy-related 16-like 1	2.55	0.02689
tr|Q7TNA8|Q7TNA8_RAT	L-lactate dehydrogenase	2.61	0.02465
sp|P56374|CRBA4_RAT	Beta-crystallin A4	2.65	0.002346
sp|P34900|SDC2_RAT	Syndecan-2	2.7	0.003389
sp|P17078|RL35_RAT	60S ribosomal protein L35	2.73	0.04972
tr|O35515|O35515_RAT	C-met/hepatocyte growth factor receptor	2.89	0.001192
sp|P60572|PANX3_RAT	Pannexin-3	2.99	0.001516
tr|F1LUV9|F1LUV9_RAT	Neural cell adhesion molecule 1	3	0.006539
sp|O08722|UNC5B_RAT	Netrin receptor UNC5B	3.13	0.00363
tr|A0A0G2K8V0|A0A0G2K8V0_RAT	Protein tyrosine phosphatase, receptor type, M GN=Ptprm	3.77	0.0115
tr|Q58NB7|Q58NB7_RAT	Retinoic acid receptor responder (Tazarotene induced) 1	4.08	0.000543
tr|B1H2A3|B1H2A3_RAT	Bromodomain PHD finger transcription factor	0.26	0.003812
sp|Q9EQH5|CTBP2_RAT	C-terminal-binding protein 2	0.4	0.006807
tr|Q63910|Q63910_RAT	Alpha globin	0.41	0.01095
tr|Q5EBA5|Q5EBA5_RAT	Lipin 3	0.46	0.00009122
tr|A0A0G2JZB7|A0A0G2JZB7_RAT	Neuron navigator 3	0.48	0.00609
sp|A0A0G2K0D3|LMOD1_RAT	Leiomodin-1	0.48	0.0462
sp|P06907|MYP0_RAT	Myelin protein P0	0.49	0.00899
sp|O88831|KKCC2_RAT	Calcium/calmodulin-dependent protein kinase kinase 2	0.5	0.03585
sp|Q5XI50|MARH7_RAT	E3 ubiquitin-protein ligase MARCH7	0.51	0.02382
sp|P19527|NFL_RAT	Neurofilament light polypeptide	0.51	0.00348
tr|D3ZDK4|D3ZDK4_RAT	Angiopoietin-like 7	0.51	0.001431

tr|A0A0G2K1L0|A0A0G2K1L0_RAT	Tenascin C	0.53	0.01641
sp|P17988|ST1A1_RAT	Sulfotransferase 1A1	0.53	0.02154
sp|Q6PCT8|DHSD_RAT	Succinate dehydrogenase [ubiquinone] cytochrome b small subunit, mitochondrial	0.54	0.04886
tr|D3ZK72|D3ZK72_RAT	Keratin-associated protein 16-5	0.56	0.002354
sp|Q08290|CNN1_RAT	Calponin-1	0.56	0.02882
sp|P04638|APOA2_RAT	Apolipoprotein A-II	0.57	0.02191
tr|A0A0G2JVN7|A0A0G2JVN7_RAT	Oogenesin 1	0.57	0.02633
tr|D3ZKQ0|D3ZKQ0_RAT	Nudix (Nucleoside diphosphate linked moiety X)-type motif 15	0.57	0.0007321
sp|O88801|HOME2_RAT	Homer protein homolog 2	0.58	0.006055
tr|Q5PQK3|Q5PQK3_RAT	Tripartite motif-containing 42	0.58	0.01908
sp|P80432|COX7C_RAT	Cytochrome c oxidase subunit 7C, mitochondrial	0.58	0.03041
sp|Q63228|GMFB_RAT	Glia maturation factor beta	0.58	0.004609
sp|Q6MG12|CF136_RAT	Uncharacterized protein C6orf136 homolog	0.58	0.02408
tr|D3ZIA7|D3ZIA7_RAT	Anterior gradient 2 (Xenopus laevis)	0.58	0.02767
tr|E9PTU4|E9PTU4_RAT	Myosin-11	0.59	0.01848
sp|P04916|RET4_RAT	Retinol-binding protein	0.6	0.001988
sp|Q566C7|NUDT3_RAT	Diphosphoinositol polyphosphate phosphohydrolase 1	0.6	0.002305
sp|P46844|BIEA_RAT	Biliverdin reductase A	0.6	0.0007787
sp|P0C089|PTPM1_RAT	Phosphatidylglycerophosphatase and protein-tyrosine phosphatase 1	0.6	0.01126
tr|Q6AYC0|Q6AYC0_RAT	HAUS augmin-like complex, subunit 4	0.6	0.03664
tr|G3V6A9|G3V6A9_RAT	Microfibril-associated protein 4	0.6	0.002131
sp|Q5XIE1|THEM6_RAT	Protein THEM6	0.6	0.01505
sp|Q5XIL3|RPC3_RAT	DNA-directed RNA polymerase III subunit RPC3	0.61	0.003253
tr|A0A0U1RRX6|A0A0U1RRX6_RAT	5-demethoxyubiquinone hydroxylase, mitochondrial (Fragment)	0.61	0.01683
sp|Q5BJT4|TXD15_RAT	Thioredoxin domain-containing protein 15	0.61	0.0008456
tr|Q5XIH1|Q5XIH1_RAT	Asporin	0.61	0.000005075
tr|A0A0G2JV49|A0A0G2JV49_RAT	Protein phosphatase 6, regulatory subunit 2	0.61	0.01109
tr|M0R5G7|M0R5G7_RAT	Keratin-associated protein 22-2	0.61	0.0173
tr|D3ZML4|D3ZML4_RAT	Similar to RIKEN cDNA	0.62	0.007883
tr|Q7TNX2|Q7TNX2_RAT	Liver regeneration-related protein	0.62	0.002522
tr|B1H271|B1H271_RAT	Mitochondrial coenzyme A transporter SLC25A42	0.62	0.001657

tr|D3ZDF3|D3ZDF3_RAT	MRV integration site 1 homolog	0.63	0.02353
tr|D3ZCS4|D3ZCS4_RAT	IQ motif-containing GTPase-activating protein 3	0.63	0.0006522
tr|D4A559|D4A559_RAT	Dematin actin-binding protein	0.63	0.0008044
sp|Q5PQN7|LZIC_RAT	Protein LZIC	0.64	0.003502
tr|A0A0G2JW50|A0A0G2JW50_RAT	Phosphatidylinositol transfer protein, membrane-associated 2	0.64	0.005186
sp|Q9EQT5|TINAL_RAT	Tubulointerstitial nephritis antigen-like	0.64	0.01697
tr|D3ZN64|D3ZN64_RAT	Collagen type XXVIII alpha 1 chain	0.64	0.01006
tr|D3ZIL6|D3ZIL6_RAT	Enoyl CoA hydratase domain-containing 2	0.64	0.01343
tr|D3ZC55|D3ZC55_RAT	Heat shock 70kDa protein 12A	0.64	0.0004399
tr|F1M8I6|F1M8I6_RAT	Homeodomain-interacting protein kinase 1	0.64	0.03932
tr|B1H257|B1H257_RAT	BLOC-1-related complex subunit 5	0.64	0.009142
tr|A0A0G2JST5|A0A0G2JST5_RAT	Elastin	0.64	0.004676
tr|F1M7B7|F1M7BRAT	6-phosphofructo-2-kinase/fructose-2	0.64	0.001324
sp|P35213|1433B_RAT	14-3-3 protein beta/alpha	0.65	0.008167
sp|Q4V8B3|MED24_RAT	Mediator of RNA polymerase II transcription subunit 24	0.65	0.004873
tr|G3V8L3|G3V8L3_RAT	Lamin A	0.65	0.003225
sp|Q9ES71|GNPAT_RAT	Dihydroxyacetone phosphate acyltransferase	0.65	0.04966
tr|D3ZCV5|D3ZCV5_RAT	Aldehyde dehydrogenase, cytosolic 1	0.65	0.01133
tr|D4ABN3|D4ABN3_RAT	Synaptojanin-1	0.65	0.03416
sp|P63031|MPC1_RAT	Mitochondrial pyruvate carrier 1	0.65	0.02843
sp|Q9Z1B2|GSTM5_RAT	Glutathione S-transferase Mu 5	0.66	0.008064
tr|Q562B7|Q562B7_RAT	Ptpn3 protein (Fragment)	0.66	0.00195
tr|A0A0G2K6I0|A0A0G2K6I0_RAT	Similar to ribosomal protein S15a	0.66	0.004194
sp|Q5DT39|KLRI1_RAT	Killer cell lectin-like receptor subfamily I member 1	0.66	0.002692
tr|A0A0G2JUU6|A0A0G2JUU6_RAT	Exosome complex component RRP45	0.66	0.02732
tr|G3V927|G3V927_RAT	Discs, large homolog-associated protein 4	0.66	0.04863
sp|P31430|DPEP1_RAT	Dipeptidase 1	0.66	0.03623
tr|D3ZS88|D3ZS88_RAT	Activating transcription factor 7-interacting protein	0.66	0.03088
sp|P14669|ANXA3_RAT	Annexin A3	0.66	0.04388

Table S3. Significant difference protein in Control-VS-10-30 kD samples.

Protein Number	Protein Description	Average Ratio	P value
sp|P07897|PGCA_RAT	Aggrecan core protein	1.51	0.01236
sp|Q9ERB4|CSPG2_RAT	Versican core protein	1.52	0.04654
tr|D4A4H5|D4A4H5_RAT	Stromal cell derived factor 2	1.52	0.03901
sp|Q8CH87|GCNT3_RAT	Beta-1,3-galactosyl-O-glycosyl-glycoprotein beta-1,6-N-acetylglucosaminyltransferase 3	1.52	0.04185
sp|P62083|RS7_RAT	40S ribosomal protein S7	1.53	0.04183
tr|D3ZIK0|D3ZIK0_RAT	Glucuronic acid epimerase	1.53	0.04154
tr|D3ZYI7|D3ZYI7_RAT	Olfactory receptor	1.54	0.007371
tr|F1M065|F1M065_RAT	Junctional adhesion molecule 2	1.56	0.04396
tr|A1L1M0|A1L1M0_RAT	Protein kinase, cAMP-dependent, catalytic, alpha	1.56	0.04614
sp|P47853|PGS1_RAT	Biglycan	1.56	0.002068
sp|Q9QX67|DAP1_RAT	Death-associated protein 1	1.58	0.0319
sp|P54001|P4HA1_RAT	Prolyl 4-hydroxylase subunit alpha-1	1.58	0.0003483
sp|P70560|COCA1_RAT	Collagen alpha-1(XII) chain	1.59	0.01162
sp|Q66H78|PXYP1_RAT	2-phosphoxylose phosphatase 1	1.59	0.03026
tr|Q6IFW1|Q6IFW1_RAT	RCG34348	1.63	0.02319
sp|P60572|PANX3_RAT	Pannexin-3	1.63	0.04626
sp|P20909|COBA1_RAT	Collagen alpha-1(XI) chain	1.63	0.002578
sp|P34900|SDC2_RAT	Syndecan-2	1.64	0.01577
tr|A7LKE6|A7LKE6_RAT	R-spondin 3	1.64	0.0223
tr|Q5XIQ8|Q5XIQ8_RAT	Hexosyltransferase	1.65	0.02586
sp|Q62632|FSTL1_RAT	Follistatin-related protein 1	1.7	0.01388
sp|Q6IE14|TM11L_RAT	Transmembrane protease serine 11B-like protein	1.71	0.004908
tr|D3Z9Z7|D3Z9Z7_RAT	Collagen beta(1-O)galactosyltransferase 2	1.71	0.005381
sp|Q62751|IREB2_RAT	Iron-responsive element-binding protein 2	1.78	0.03392
tr|O35515|O35515_RAT	C-met/hepatocyte growth factor receptor	1.8	0.004598
tr|F1LND0|F1LND0_RAT	Collagen type XVI alpha 1 chain	1.86	0.02595
tr|A0A0G2K7C2|A0A0G2K7C2_RAT	Kinesin family member 20B	1.9	0.02059
tr|Q58NB7|Q58NB7_RAT	Retinoic acid receptor responder	1.96	0.001302
tr|A0A0G2K8V0|A0A0G2K8V0_RAT	Protein tyrosine phosphatase,	2.07	0.001518

sp|P01143|CRF_RAT	Corticoliberin	2.27	0.0001548
tr|D4ACD9|D4ACD9_RAT	Protein tyrosine phosphatase domain containing 1	2.35	0.03423
tr|Q7TNA8|Q7TNA8_RAT	L-lactate dehydrogenase	2.57	0.03264
tr|M0R7M4|M0R7M4_RAT	RAD51-associated protein 2	3.6	0.00612
tr|A0A096MJI4|A0A096MJI4_RAT	Uncharacterized protein (Fragment)	4.38	0.04871
tr|B1H2A3|B1H2A3_RAT	Bromodomain PHD finger transcription factor	0.23	0.003362
tr|A0A0G2JSH5|A0A0G2JSH5_RAT	Serum albumin	0.41	0.000387
tr|Q9JKL3|Q9JKL3_RAT	Apoptosis regulator BAX	0.48	0.02065
tr|Q5PQK3|Q5PQK3_RAT	Tripartite motif-containing 42	0.5	0.005958
tr|D3ZVP9|D3ZVP9_RAT	Solute carrier family 4 member 11	0.52	0.008403
sp|P04638|APOA2_RAT	Apolipoprotein A-II	0.53	0.01744
tr|A0A0G2JVN7|A0A0G2JVN7_RAT	Oogenesin 1	0.55	0.02082
sp|Q4V7F2|CREL1_RAT	Protein disulfide isomerase Creld1	0.56	0.0001455
tr|Q63910|Q63910_RAT	Alpha globin	0.56	0.03798
tr|Q63011|Q63011_RAT	Zero beta-globin	0.56	0.04836
sp|Q99MB4|CBWD1_RAT	COBW domain-containing protein 1	0.58	0.03405
tr|D3ZIA7|D3ZIA7_RAT	Anterior gradient 2 (Xenopus laevis)	0.58	0.02856
tr|F1M1C9|F1M1C9_RAT	Phosphorylase b kinase regulatory subunit	0.59	0.04483
tr|D3ZKQ0|D3ZKQ0_RAT	Nudix (Nucleoside diphosphate linked moiety X)-type motif 15	0.59	0.00001754
sp|P17988|ST1A1_RAT	Sulfotransferase 1A1	0.6	0.001972
tr|M0R9U2|M0R9U2_RAT	Ig-like domain-containing protein	0.6	0.02211
tr|D3ZPE2|D3ZPE2_RAT	Family with sequence similarity 222	0.61	0.01745
tr|Q6MGA0|Q6MGA0_RAT	MHC class II antigen	0.62	0.01201
tr|A0A0G2JSW3|A0A0G2JSW3_RAT	Globin a4	0.63	0.04567
tr|Q5EBA5|Q5EBA5_RAT	Lipin 3	0.64	0.03012
sp|P31430|DPEP1_RAT	Dipeptidase 1	0.64	0.03147
sp|O88801|HOME2_RAT	Homer protein homolog 2	0.65	0.01753
tr|G3V927|G3V927_RAT	Discs, large homolog-associated protein 4 (Drosophila)	0.65	0.04231

sp|P04916|RET4_RAT	Retinol-binding protein	0.66	0.001538
sp|P19527|NFL_RAT	Neurofilament light polypeptide	0.66	0.00001774
